# Community Health Teams: a qualitative study about the factors influencing the decision-making process

**DOI:** 10.1186/s12913-023-09423-6

**Published:** 2023-05-10

**Authors:** Lisa W. Natkin, Eline van den Broek-Altenburg, Jamie S. Benson, Adam Atherly

**Affiliations:** 1 University of Vermont, The Robert Larner College of Medicine, Burlington, VT 05405 United States; 2 Department of Health Administration, Virginia Commonwealth University, Richmond, VA 23284 United States

**Keywords:** Community health, Decision making, Resource allocation, Community health teams, Healthcare reform, Value-based care

## Abstract

**Background:**

The purpose of this study was to explore the factors influencing how individual Community Health Teams (CHTs) make decisions about what services to offer and how to allocate their resources.

**Methods:**

We conducted thirteen semi-structured interviews with all 13 CHTs program managers between January and March, 2021. We analyzed interviewees descriptions of their service offerings, resources allocation, and decision-making process to identify themes.

**Results:**

Four major themes emerged from the interview data as factors influencing community health team program managers’ decision-making process: commitment to offering high-quality care coordination, Blueprint’s stable and flexible structure, use of data in priority setting, and leveraging community partnerships and local resources.

**Conclusions:**

Community-based CHTs with flexible funding allowed programs to tailor service offerings in response to community needs. It is important for teams to have access to community-level data. Teams are cultivating and leveraging community partners to increase their care coordination capacity, which is focus of their work. CHTs are a model for leveraging community partnerships to increase service capacity and pubic engagement in health services for other states to replicate.

## Background

Community Health Teams (CHTs) have been widely touted as a population health approach to improve health outcomes, health equity, and reduce healthcare spending, and are particularly effective at reducing health disparities [1–3]. While traditional patient care happens within the walls of physician’s offices, the work of the CHTs bridges the gap between clinical care and daily lifestyles. Yet relatively few states have developed and funded statewide networks of CHTs. Funding CHTs is challenging, and the funding model directly influences the role CHTs play in the healthcare ecosystem [4]. For example, CHTs funded as integrated members of primary care teams necessarily focus services on covered patient populations; conversely, CHTs funded as independent, community-based teams theoretically focus on broader population health and health equity issues. Despite the potential advantages of community-based CHTs, primary care based teams are more common because of the ease in identifying potential cost savings to justify funding [5]. Care provided in these kinds of patient-centered, accountable care settings has been found to be associated with lower total expenditures [6].

One example of a successful statewide community-based CHT model is Vermont, which has sponsored a statewide network of CHTs for almost two decades through the Blueprint for Health initiative (Blueprint). CHTs in Vermont are an integral part of a statewide care transformation model supported by Blueprint, which emphasizes community-led strategies for improving health and well-being. Blueprint ensures that there is at least one CHT in each of the state’s 13 Health Service Areas (HSAs) to provide support services for the population of patients and connect individuals with each region’s available resources. CHTs enable access to individual care coordination, substance use disorder treatment, dietary and nutrition services, and counseling, among other social and economic services [7]. These CHTs work with both medical and community providers to establish regional health priorities, support learning collaboratives, and work to improve quality of services for health and well-being.

Additionally, Vermont received a waiver in 2018 from the Centers for Medicare and Medicaid Innovation (CMMI) to create the Vermont All-Payer Model (VAPM). The VAPM is unique nationally in that the Medicare, Medicaid and commercial insurance contracts are allowed to pay a prospective actuarially determined “all-inclusive population-based payment” monthly for all anticipated inpatient, hospital outpatient and professional services for the attributed beneficiaries. The VAPM is part of an ambitious effort to fundamentally alter the misalignment of payment incentives across all payers and create an environment where care providers can shift their focus from revenue generation to population health targets [8]. The VAPM builds on the same CHT structure established by Blueprint, creating a new potential source of funding for CHTs that could be replicated nationally. The VAPM allows the creation of all-payer Accountable Care Organizations (ACO); there is currently one operating in Vermont (“OneCare”), which covers approximately half of the state’s population. How a CHTs program with flexibility and a community focus, interacts with a centralized health system focused initiative is unclear.

Of interest is how CHTs set priorities for different populations and services. To date, little is known about priority setting for community based CHTs, although this is the CHT model that is viewed as optimal for population health. This paper responds to that gap by studying how CHT program managers make decisions about which services to offer and how to allocate their resources, such as staffing composition. This paper provides a window into the factors that influence the decision-making process in a community-based CHT model [9, 10]. Understanding which factors influence community health program managers’ team composition, structure, and care coordination services provides lessons for how other states to replicate Vermont’s approach to CHTs.

## Methods

Data collection activities were part of a Robert Woods Johnson Foundation grant evaluating the effect of combining a global all-payer reimbursement with Community Health Teams responsible for coordinating care and services delivery between the medical, social services, and public health sectors on system alignment, health, access to healthcare and healthy equity. To identify factors which influenced CHT leaders’ decision-making process, we interviewed CHT program managers. Data was thematically analyzed to identify patterns related to the following research question: What factors influence CHT leaders’ decision-making process for resource allocation and service offerings?

The interview protocol for CHT program managers was developed in collaboration with Blueprint leaders and piloted with a community health leader. To aid interviewee recruitment, a member of the Blueprint leadership team told all CHT program managers to expect an email from our research team inviting them to participate in a 1-hour virtual recorded interview. Through quota sampling methods, our sample included all thirteen CHT program managers to sufficiently gather information about factors influencing leaders’ decision-making process [11]. Program managers were invited to include other CHT team members in the interviews: some program managers did, and some did not. In total, all CHT program managers representing the thirteen HSAs accepted our invitation to participate in a 1-hour semi-structured interview conducted between January 11 and March 03, 2021.

We asked interviewees about their current service offerings, factors that influenced their decision-making process, if their decisions were data-driven, and if so what types of data were consulted (see Table 1 for interview topics and questions). Prior to the interviews, interviewers reviewed recent annual reports and relevant websites to get insights on the thirteen HSAs’ program offerings, enabling them to probe interviewee answers as needed. Our multidisciplinary team consisted of university-based behavioral health researchers and faculty. The three-member research team conducting the interviews consisted of two females with PhDs and one male research specialist. One female researcher is an Assistant Professor of Radiology with expertise in population health science, health services research, and decision analysis. The other female was a research analyst in the Division of General Internal Medicine Research with expertise in qualitative methods. The male researcher was a research specialist in the Department of Radiology and the Department of Surgery, Division of Acute Care Surgery, with expertise in health systems assessment and decision analysis. When possible, two members of the research team participated in the interviews and debriefed afterwards. The researcher with expertise in qualitative methods led the analysis process.

**Table 1:** Interview topics and corresponding sample interview questions

Interview Topics	Sample Questions
**CHT’s Current Service Offerings**	• Please describe what community health issues you are currently focusing on in your service area. • In your experience, which of your programs have been successful in improving people’s engagement in their own health?
**Factors in Decision Making Process**	• As a local Program Manager of the Blueprint, please describe your decision-making process for prioritizing how you allocate funding resources. How do you decide FTE allocations for CHT staff? • How do you decide what type of CHT staff to hire, such as social workers, dietitians, RNs, panel managers, behavioral health, and care coordinator? • What factors do you consider in your decision-making process about resource allocation?
**Informed Decision Making**	• Who do you typically consult with when making decisions about service offerings? • Please describe how evaluation data is gathered to assess if your services are successful. • Describe a time you decided to shift your resource allocation. What factors informed your decision-making process?

Interview recordings were transcribed and imported into Dedoose, our qualitative research management software [12]. Our thematic analysis entailed a combination of deductive and inductive approaches [13]. Using a deductive approach, a code framework was developed informed by the interview topics and initial review of the transcripts [13]. This *a priori* code framework was imported into Dedoose to provide a structure to organize analysis. Subsequent analysis of transcripts was guided, but not confined, by the preliminary code framework. Using an inductive approach, emerging codes were added as the researcher carefully analyzed the interview transcripts continuing to refine and reorganize the codebook [14, 15].

Through processive cycles of coding, the recurring patterns across congruent codes informed the subthemes [13], Identified subthemes were closely scrutinized for alignment to ensure they were representative of the data. Analysis began at the individual HSA level to establish the context and characteristics of each region, followed by an analysis across all HSAs [16]. To assist with cross-region comparisons, a table outlining key characteristics and themes was created. Project team members and partners provided feedback on several iterations of emerging categorizations to collapse and refine themes. The transcripts were not provided to the interviewees out of respect for their limited time.

## Results

Four major themes emerged from the interview data as factors influencing CHT program managers’ decision-making process: commitment to offering high-quality care coordination, Blueprint’s stable and flexible structure, use of data in priority setting, and leveraging community partnerships and local resources. The major themes represent what was most prominent in the data in terms of strength and salience in relation to our research question; all are related and overlap with one another. Each of the four themes are presented in Table 2 and discussed below.

**Table 2:** Key Themes Influencing Program Managers Priority Setting and Resource Allocation

Theme	Subtheme	Illustrative Quotations
Blueprint’s Stable and Flexible Structure	Blueprint enables local teams to create own structure and services	“The beauty of Blueprint is, it is quite flexible in terms of how we deploy that funding and turn it into staff. Their emphasis is at a community level, we are responsive to the community needs.”
	Investment in building team capacity	“I think that the Blueprint has been the bedrock. There was a series of learning collaboratives that brought national experts to build capacities and understand best practices in care coordination. It is being able to work from a grounding of research instead of what just feels good. There has been a lot of latitude in how you develop who you hire for the staffing through the Blueprint and in the care coordination work.”
Commitment to offering High Quality Care Coordination	Individualized care coordination for all patients	Engaging them with what they feel is most meaningful to them at that time. We want to figure out what engages them. What they need in that moment so they can see the value to working with someone and getting that support to navigate systems. We can help patients take these next steps of what the provider is asking them to do and really support them getting the resources they need to make that happen.”
	Access to supplemental funding for staff and programs	“The physician that is in the Emergency Department is a shared position between our FQHC and the hospital. That position came out of a community conversation about needing more support for folks that come to the ED. Maybe patients are really there for social needs or they need support in getting connected to follow-up care. We have positions that were a decision from the community and responsive to a community need. That is certainly emphasized in our Blueprint contract.”
Use of Data in Program Priority Setting	Needs of the community and patients	“I was looking at the Youth Risk Behavior Survey and resiliency was identified as one of the priorities. The Health Department and our designated agency, came together to spearhead the Okay Resiliency Campaign. Different community partners started getting involved to identify different tools to help parents support resiliency in the household. During the past two years, there has been a network of volunteers that have helped create a curriculum for parents and in schools.”
	Data-driven decision making	“Previously, we used the Blueprint profiles. We shared those with the practices. But those profiles have since been retired. That definitely presented this vulnerability for our team, so my team just recently created this Blueprint Data Brief, where we pulled out our most important information that would attest to the work that we are doing. We are still refining those measures that we chose because we want it to be reproducible data every month.”
Leveraging Community Partnerships and Local Resources	Strength of community network	“It is important for us to also have a strong infrastructure and Community Health network. In recognizing that we have limited finances, we try to maximize the resources of the community working together. We recognize the strengths of own community partners and have an infrastructure so that we have access to them.”
	Availability of local resources and services	“We have no homeless shelter. We have a gap in that area compared to the rest of the state. Obviously, there are risk factors for people that our care team works with. We have nurses that are highly trained not working to the top of their license because they are making ride arrangements or filling out their housing application. It is a crazy use of time for a nurse, but that is the best that we can do with the staffing we have.”

### Commitment to offering high quality care coordination

The first theme was the CHTs’ focus on individual patient care coordination. Many CHT program managers shared that their care coordinator’s work centers around what is most important to the patient, many times asking the patient about what they hope to address through these services. Interviewees described how this strategy cultivates trusting relationships with patients, and is an essential step to increase patient engagement for behavioral health modifications. A care coordinator describes their approach as *“engaging them [the patient] with what they feel is most meaningful to them at the time. What they need in that moment to see the value to working with someone to get support.”* (Interview #PM9) Many interviewees shared stories of helping patients address barriers to accessing services, such as transportation or communication with their primary providers and specialists. *“We help patients take those next steps of what the provider is asking them to do and support them getting the resources they need to make it happen,”* (Interview #PM9) one program manager noted.

Many interviewees shared examples of care coordinators being funded by hospitals, FQHCs, mental health agencies, and community partners or practices, because the patient need exceeded the Blueprint-funded team’s capacity. Having additional care coordinators helps support CHTs’ ability to meet patient care coordination needs. Regardless of funding sources, many teams described meeting regularly to share resources and stay informed about community partners’ offerings to accomplish more as extended teams. A few health service areas were able to secure grant funds for positions, enabling coordination of social services. For example, stationing a care coordinator at the emergency department to rapidly link patients in crisis to social services is responsive to the community’s needs. Another program manager explained *“they receive a grant to purchase grocery store gift cards and they have been a really valuable way to engage people by meeting an immediate need.”* (Interview #PM9).

To ensure care coordination services are utilizing best practices, local and state Blueprint leaders host trainings and learning collaboratives. One program manager underscored their commitment to investing in their staff by offering *“funding to support training to continue to build their skillset.”* (Interview #PM9) They invest in building team capacity, and support staff in earning new licenses and skills. This ability and commitment to building capacity within their team influences staffing decisions, impacting resource allocation. HSA staff composition also responds to the changes in community needs and healthcare policies. For example, one CHT program manager has increased the number of social workers on their team due to patients’ need for services.

### Blueprint’s stable and flexible structure

The next salient theme is related to the flexibility the State of Vermont’s Blueprint for Health, which allows all 13 HSAs to create their own unique organizational structures, funding arrangements, and services. Teams work with their administrative entities and participating practices to identify the best funding arrangements for their community. This ability to be responsive to local context has resulted in a variety of structures. Some community health staff are hired directly by the administrative entities but work onsite at the primary care practices. Some staff are hired directly by and work at the hospital, receiving referrals for care coordination from primary care practices. Funds may also be “passed through” to practices so they can hire their own care coordination staff. A few CHTs have arrangements with their designated mental health agency to hire and manage staff working in the practices.

Blueprint funding is dedicated to CHT staff salaries, so hospitals and practices do not have to pay CHT staff. This funding results in care coordination services being free to patients, removing financial barriers to access. As a program manager noted, *“there is no bill that gets generated for our services, so money does not have to be factor in whether they agree to our services or not.”* (Interview #PM4) This financial freedom also allows care teams the flexibility to respond to patients’ needs. A few program managers explained that they will meet a patient where it is convenient for them, such as their home or a grocery store. This also allows care coordination services to reach typically underserved patients. An example of this was shared,*“we have a health coach that will go grocery shopping with people to help them pick things that are better for their disease.”* (Interview #PM13) Care coordinators frequently tailor their services to meet individual patients’ needs.

### Use of data in program priority setting

Another prominent theme shared by CHT program managers was how data about their HSA’s unique context and set of community needs influenced their priority setting. Each HSA conducts Community Health Needs Assessments (CHNA) to gather data identifying priority areas. CHNAs are sometimes supplemented with data from the Behavioral Risk Factor Surveillance System (BRFSS) and Youth Risk Behavior Surveillance System (YRBSS). Community needs inform staffing decisions, such as which type of credentialed staff are in highest demand. A program manager explained, *“we knew we were going to need at least a nurse care coordinator and a dietician because of the high rate of obesity and diabetes in our area.”* (Interview #PM7) In addition, teams gather referral data to inform program improvement.

Many program managers described a strong desire to make data-driven decisions, but some were challenged by gathering utilization data or not receiving data from Blueprint. Blueprint used to create “data profiles” for each practice which were helpful tools to communicate progress, however the practice profiles are no longer provided due to funding cuts. Now, a few CHTs are creating their own data reporting systems and dashboards to inform their decision-making and communicate progress. One CHT team shared that they created a quality dashboard to present at meetings with community partners reviewing data related to diabetes, hypertension, food insecurity, substance use disorder. Some program managers shared they would like to consult data more consistently when making decisions, but lack the resources and capacity.

### Leveraging community partnerships and local resources

The final theme influencing priorities and service offerings was the ways in which CHT members leveraged their community partnerships and local resources. Program managers described the unique makeup of staff credentials in their HSA based on local need and availability of other services. Program managers explained how they consider which services are available in their communities and attempt to fill in gaps for needed services with their staff and offerings. For example, if behavioral health services are scarce in a region, the program manager might prioritize hiring behavioral health providers. One program manager shared that their HSA does not have a homeless shelter, so they prioritize working with the homeless population and offering services for them.

The Blueprint program requires CHTs to have a “community collaborative,” which are work groups accountable for meeting the population health goals. One program manager explained how the process works for their collaboratives, *“we identified the priority for the four work groups starting with the data from the CHNA, going into the public arena, identified partners who are willing work and we have 99 partners from different agencies and groups that are integrated into the efforts.”* (Interview #PM6) The community collaboratives are comprised of leaders from local organizations who understand the current health status of the community and work together to design actionable goals for improvement. A program manager explains, *“the leaders are at the table who have all decided to commit to working together in our region to improve health.”* (Interview #PM12) Most program managers shared how the collaboratives are an important networking vehicle for cultivating partnerships and exchanging information and resources. One program manager elaborated that the collaboratives are a place where *“people share resources, problem solve on care coordination issues, brainstorm on what will work.”* (Interview #PM6).

Community partners donate their time to the CHT’s, working collectively as part of their mission. As a program manager illustrates, *“the wonderful thing is that they are funding their work through their own different agencies.”* (Interview #PM6) Blueprint funding supports staff salaries, so leveraging community partnerships is how many teams make community programming happen. In many cases, CHTs have been able to multiply their impact through these partnerships. For example, one program manager shared that they noticed in their YRBSS that many students showed low resiliency, so they partnered with the Health Department and their designated agency to create a resiliency campaign. They worked with community partners to create tools and curricula for parents and teachers. The strength of community partnerships varies among the HSAs: some are extremely active with a broad scope focused on addressing community health needs, while others are less engaged with a narrower scope. The strength of these community networks influences resource allocation and priority setting.

### Influence of the COVID-19 pandemic

Given the timing of our interviews with CHT members, interviewees shared many ways the COVID-19 pandemic influenced their work. In some HSAs, staff helped with COVID-19 testing and vaccine administration. Others called their patients to check in and make sure they were getting the medications and care they needed. Self-management programs switched to being offered virtually, which has increased access and participation. Nationally, telehealth has increased helping to address barriers to care for some patients [17, 18].

Thanks to their strong community networks, CHTs were able to directly support the local pandemic response. One program manager articulated, *“we are trying to support the development of capacity to better coordinate care, keep those relationships going and be in place to address things as they arise, so with COVID we easily transitioned to help set up systems to address food insecurity and get health information out.”* (Interview #PM10) A few CHT program managers shared their belief that the pandemic is increasing the need for mental health services particularly among youth. An interviewee illustrates, *“I am concerned about the far-reaching implications of this, especially on our pediatric population.”* (Interview #PM4).

### VAPM and CHT priorities

The establishment of the VAPM has influenced the work of CHTs in many ways. Though the VAPM is not directly funding the CHTs, interviewees shared many ways CHTs are relied upon to support the population health goals of the VAPM. The VAPM ultimately must control healthcare spending, so their focus is on medically high-risk patients. In contrast, the CHTs are more focused on the social determinants of health across the entire HSA population. This means the VAPM priority is high-cost patients, while historically CHTs aid any patient who needs to be connected to a community service. The VAPM also focuses on attributed lives (approximately half the state’s population), which means they only fund services to the subset of the population that is attributed to the ACO; in contrast the CHTs provide services based on need rather than eligibility.

VAPM’s payer arrangements also require that a licensed care coordinator serves certain patients to qualify for payments, which has changed some CHTs’ resource allocation. One CHT leader shared, *“we are hiring another clinical care coordinator and we need that person to have a license to help manage the ACO attributed population.”* (Interview #PM9) The CHTs’ administrative entities are paid by the VAPM based on services rendered rather than receiving fixed funding like Blueprint. One program manager explains,

*“Blueprint funding is very clear, we know how much we are getting on a quarterly basis so I can plan my full-time equivalents around that, compared to VAPM funding is value-based so it is not well defined. I think it goes to the hospital’s bottom line and does not translate into care coordination services.”* (Interview #PM1)

This creates a new administrative challenge for the CHTs to record and bill for services. Taken together, the VAPM implicitly pushes the CHTs to change their community focus, financial structure, and service offerings.

## Discussion

In this study, we sought to understand the factors that influence CHTs’ decisions about what services to offer and how to allocate their resources. We studied a community based CHT program in the state of Vermont. The CHTs were paid a fixed amount per year and given flexibility in the services offered and the populations prioritized.

The results from our qualitative study revealed the following influential factors: (1) Blueprint’s flexible structure, (2) commitment to offering high-quality care coordination, (3) use of data, and (4) strength of community partnerships. Interviewees’ perceptions and experiences offer insights into their decision-making process for resource allocation and service offerings, serving as a potential model for other states.

The flexibility of Blueprint’s funding and their empowerment of local teams enables them to provide care coordination to all patients. This flexibility allows teams to make decisions about how to allocate their resources directly in response to community needs. Many CHTs believe their collaborations provide economies of scale as well as additional funding for flexible projects. They cultivate and leverage community partners to increase their care coordination capacity. Community partners help to determine community initiatives and programs. Blueprint and CHT program managers invest in building their teams capacities, and work to multiply their impact by increasing coordination and cross-communication. The availability of professional development opportunities and trainings influences how teams are able to allocate their resources to meet dynamic patient needs.

CHTs gathers and consult data about community needs and utilization of services to set priorities for programing and staffing decisions. The Blueprint has an important task in feeding the CHTs relevant data about changing demographics, environmental circumstances, funding mechanisms and other relevant factors to inform their decision making. CHTs also need to report data to their centralized organizations to inform the effect of existing programs. The two-way flow of data is impeded by the lack of a standardized reporting process. Further, CHTs find themselves between two competing structures: one capitated and value-based (Blueprint), and the other capitated, but retaining the limitations and structure of a fee-for-service model (VAPM). This duality creates friction between the priorities of the community, the State, and the funder.

Sustainable funding is a primary challenge for CHTs in the context of the VAPM. The CHTs so far have been flexible in finding alternative funding from a multitude of sources, including community partners, grants, and partners in the healthcare system. But the VAPM represents a unique opportunity for growth and sustained, stable funding – while also presenting new challenges. The additional funding provides the opportunity to expand services yet comes with restrictions and additional administrative burden. In theory, the VAPM structure should create alignment of incentives for population health between the healthcare sector and CHTs, while also providing additional funding for expansion of CHTs. Currently however, the funding is routed through OneCare for their attributed population and supports VAPM’s uniform statewide goals; in contrast to the CHTs non-uniform, community driven goals for the *entire* population. The differences in focus between the VAPM, OneCare and the CHTs in patient populations and goals may be creating a misalignment between entities who should be partnering to foster a healthy population.

## Conclusions

CHTs are a replicable model for leveraging community partnerships to increase service capacity and public engagement in health services for other states. Community based CHTs with flexible funding can work closely with their communities to develop programs that are responsive to community needs. The CHTs gather community needs data, build partnerships and coalitions with community partners and then design programs and hire staff to fit the needs and priorities of their local communities.

## Data Availability

Given the identifiable nature of the interview transcripts, data will not be made available publicly. The interview protocol and transcripts created during the current study may be reasonably requested from the corresponding author.

## List of abbreviations

CHTs: Community Health Teams
CMMI: Centers for Medicare and Medicaid Innovation
VAPM: Vermont All-Payer Model
ACO: Accountable Care Organizations
CHNA: Community Health Needs Assessments
